# An Entropic Approach to Estimating the Instability Criterion of People in Floodwaters

**DOI:** 10.3390/e23010074

**Published:** 2021-01-06

**Authors:** Zhongfan Zhu, Yongpeng Zhang, Lufeng Gou, Bo Pang

**Affiliations:** Beijing Key Laboratory of Urban Hydrological Cycle and Sponge City Technology, College of Water Sciences, Beijing Normal University, Beijing 100875, China; 201921470031@mail.bnu.edu.cn (Y.Z.); 202021470007@mail.bnu.edu.cn (L.G.)

**Keywords:** flooding, people, instability, criterion, entropy

## Abstract

People are always susceptible to a loss of stability in urban floodwaters that leads to serious casualties. Thus, the safety criterion for the instability of people in floodwaters must be determined. In this study, the hydrodynamic criterion of the instability of people in floodwaters in terms of the incipient velocity and water depth is derived using the probability method based on Shannon entropy theory. The derived model can characterize variations in the incipient velocity of people in floodwaters with respect to the inundating water depth. Furthermore, a comparison with seven experimental datasets available in the literature shows the validity of the proposed entropy-based model considering data scattering. A sensitivity analysis of the derived model to some of the incorporated parameters was performed, and the qualitative results are in accordance with our understanding of the physical mechanism of the instability of people in floodwaters. Taking the physical parameters (height and mass) of Chinese adults and children as a representative example, this study also showed the vulnerability degree of Chinese adults and children subject to floodwaters. These findings could provide a reference for administrators and stakeholders for flood hazard mitigation and flood strategy management. This study shows that an entropy-based method could be a valuable addition to existing deterministic models for characterizing the instability criterion of people in an urban flooding event.

## 1. Introduction

In the context of global climate change and rapid urbanization, many cities worldwide are experiencing consequences due to frequent flash floods (e.g., [1,2]). Taking China as an example, more than 100 cities were threatened by flash floods in China annually from 2010 to 2017 [3], and 642 cities have undertaken urgent flood-defense tasks, including 288 plain cities, 297 hilly cities, and 27 coastal cities [3,4]. Direct human deaths caused by flash floods reached 5500 per year from 1950 to 1990, 3940 from 1991 to 1999, and 1610 after 2000 in China [3]. In the summer of 2020, 70.471 million persons in 28 provinces experienced flood disasters, and 271 people died or were missing, according to statistical data published by the Ministry of Emergency Management of China, and the direct economic losses amounted to 214.31 billion RMB. Certain urban flooding events were widely reported in the media. For example, a torrential rainstorm event that occurred on 21 July 2012 in Beijing, the capital of China, affected 1.9 million people (including 79 deaths) and led to almost 11.64 billion RMB in direct economic losses (e.g., [5]). When a strong urban flood occurs, pedestrians and vehicles parked on roads experience a loss of stability, especially in low-lying areas, due to the abruptly rising water level and increasing flow velocity, and they may even be washed away by raging floodwaters, which can cause enormous losses in terms of life and property [6,7]. Therefore, it is important to investigate the safety guidelines for people and vehicles subject to urban flooding.

This study focuses solely on the safety criterion of people in floodwaters. Many studies have been performed to investigate the hydrodynamic instability of people subject to urban flooding via different methods, including mechanics-based analysis (e.g., [6,8,9]), numerical simulation techniques (e.g., [10]), laboratory flume experiments (e.g., [11,12]), and in situ observations (e.g., [13]). Flow velocity and water depth are commonly introduced as two important parameters involved in the safety criterion for flood risk assessments in previous studies. In general, three types of methods have been used to determine the instability threshold of people during flood events in the present literature.

The first kind of instability threshold is derived from simple empirical analyses based on experimental data of people during flood events. Foster and Cox [14] were the first to test the sliding instability of six children aged 9 to 13 years old in a laboratory flume, and they found that many factors are involved in the instability threshold. By examining the stability of 20 adults and one rigid body monolith in a laboratory flume, Abt et al. [11] found that the product of flow velocity and water depth appeared to be a good predictor for human instability in floodwaters, and this product also exhibits an exponentially increasing behavior with the product of the height and the weight of the human body. Takahashi et al. [15] identified the stability of three adults in a flume and focused on analyzing the drag force and friction force exerted on the human body by the incoming flow. Seven adults with a height range of 1.60–1.95 m and a weight range of 48–100 kg were tested in Karvonen et al. [12], who pointed out that the product of flow velocity and water depth has a linear mathematical relationship with the product of human height and weight. This linear function has different slopes and intercepts depending on the outer surrounding environment, including the flow condition, physical condition of the human body, contact condition between the human soles and wet ground, and lighting.

The second kind of instability threshold is based on analyses of the forces acting on a conceptual human model, and it has been adopted to simplify the pattern of flooding on the human body. Lind et al. [16] proposed three conceptual mechanical models for human toppling by considering a human body immersed in flooding water to be a circular cylindrical body, a square cylindrical body, and a cylindrical composite body. In the study of Milanesi et al. [17], the human body in a flood was conceptualized as a set of cylinders in an inclined plane, and by analyzing the force/moment equilibrium, the incipient flow condition formula for instability was derived and validated by available experimental datasets. Arrighi et al. [10] introduced a dimensionless mobility parameter for partly submerged people in incoming floodwater, and it includes flow conditions and physical characteristics of the human body and shows a linear relationship with the Froude number of the flow. These authors also discussed the variations in some forces acting on the human body using a three-dimensional numerical model.

The last kind of instability threshold method is the mechanics-based and experimentally calibrated approach (named by Kvocka et al. [18]), which was based on the innovative work of Xia and colleagues. A mechanics-based method similar to incipient sediment motion on the channel bed was used by Xia et al. [6,8] to derive the incipient velocity formulae for slipping instability and tumbling instability of the human body, and they calibrated some coefficients by conducting a series of laboratory flume experiments. The calibrated incipient velocity formulae were consistent with existing experimental datasets of real-life instability among people. Accounting for the postural adjustment of a human body in floodwater, Chen et al. [9] derived new incipient velocity formulae for human instability.

These studies have provided important physical insights into the hydrodynamic instability mechanism of people subject to urban flooding and presented applicable safety criteria for urban flood hazard assessments. However, uncertainties are associated with human instability evaluations in floodwaters because many factors play a role in triggering a loss of stability on wet ground, such as the geometrical characteristics of people during flood events, the clothing style, the degree of wear on the soles, psychological state of the tested people, the turbulent nature of the floodwater, and outer environment (for example, lighting), (e.g., [6,10]). Therefore, it is worth investigating the instability of people during urban flooding based on the probability method. In recent decades, the probability method based on information entropy has been widely applied to tackle hydrodynamic problems, such as velocity profile prediction (e.g., [19]), suspended sediment concentration distribution (e.g., [20]), and shear stress distribution (e.g., [21]). In these works, the entropy-based method showed a higher prediction accuracy for experimental data, thus indicating its potential as a valuable addition to deterministic models for dealing with hydrodynamic problems.

In this study, we attempt to derive the criterion of hydrodynamic instability for people during flood events in an urban flooding area by adopting the probability method based on the Shannon entropy theory in Section 2. The derived instability criterion is tested against seven experimental datasets available in the literature in Section 3. The sensitivity of the entropy-based model to some of the incorporated parameters is discussed in Section 4. An application of the model to map the vulnerability degree using physical parameters (height and mass) of the Chinese human body as a representative example is presented in Section 5. Finally, concluding remarks are provided in Section 6.

## 2. Entropic Model Formulation

Considering some uncertainties associated with the turbulent nature of floodwaters and the instability form of people during flood events in urban flooding, it could be reasonable to assume the incipient velocity of people during flood events U to be a random variable. Derivations of the expressions for this velocity based on the Shannon entropy theory are presented below.

The Shannon entropy function for the incipient velocity of the flooding in floodwaters U is written in terms of a continuous form as follows [22]:

(1) $$S\left(U\right)=-\int_{U_{0}}^{U_{1}}f\left(U\right)\ln\left[f\left(U\right)\right]dU$$

where S(U) is the Shannon entropy function, f(U) is the probability density function, and $U_{0}$ and $U_{1}$ are the lower and upper limits of U. The probability density function f(U) is subject to two constraints:

(2) $$\int_{U_{0}}^{U_{1}}f\left(U\right)dU=1$$

(3) $$\int_{U_{0}}^{U_{1}}Uf\left(U\right)dU=\bar{U}$$

where Equation (2) shows the probability law and Equation (3) denotes the mean constraint; here, $\bar{U}$ is the average value of observed incipient velocity values U.

In accordance with the maximum entropy principle by Jaynes [23,24,25], the probability density function f(U) should be chosen by formulating the Lagrangian function $L_{S}$:

(4) $$L_{S}=-\int_{U_{0}}^{U_{1}}f\left(U\right)\ln\left[f\left(U\right)\right]dU+\lambda_{S0}\int_{U_{0}}^{U_{1}}f\left(U\right)dU+\lambda_{S1}\int_{U_{0}}^{U_{1}}Uf\left(U\right)dU$$

where $\lambda_{S0}$ and $\lambda_{S1}$ are two Lagrange multipliers based on the Shannon entropy, differentiating its derivative with respect to f(U) and setting it to zero, finally leads to the following expression for f(U):

(5) $$f\left(U\right)=\exp\left(\lambda_{S0}-1\right)*\exp\left(\lambda_{S1}U\right)$$

Integrating Equation (5) from $U_{0}$ to U can lead to the cumulative distribution function (CDF), F(U), of U:

(6) $$F\left(U\right)=\int_{U_{0}}^{U}f\left(U\right)dU=\frac{\exp\left(\lambda_{S0}-1\right)}{\lambda_{S1}}\left[\exp\left(\lambda_{S1}U\right)-\exp\left(\lambda_{S1}U_{0}\right)\right]$$

Substituting Equation (5) into Equation (1) yields the maximum entropy function as follows:

(7) $$S\left(U\right)=-\frac{\exp\left(\lambda_{S0}-1\right)}{\lambda_{S1}}\left\{\begin{array}{l} \left(\lambda_{S0}-2\right)\left[\exp\left(\lambda_{S1}U_{1}\right)-\exp\left(\lambda_{S1}U_{0}\right)\right]\\ +\lambda_{S1}U_{1}\exp\left(\lambda_{S1}U_{1}\right) \end{array}\right\}$$

Consequently, two Lagrange multipliers, $\lambda_{S0}$ and $\lambda_{S1}$, can be obtained by solving the following nonlinear equation system that is obtained by substituting Equation (5) into Equations (2) and (3), respectively:

(8) $$\frac{\exp\left(\lambda_{S0}-1\right)}{\lambda_{S1}}\left[\exp\left(\lambda_{S1}U_{1}\right)-\exp\left(\lambda_{S1}U_{0}\right)\right]=1$$

(9) $$\frac{\exp\left(\lambda_{S0}-1\right)}{\lambda_{S1}^{2}}\left[\exp\left(\lambda_{S1}U_{1}\right)\left(\lambda_{S1}U_{1}-1\right)-\exp\left(\lambda_{S1}U_{0}\right)\left(\lambda_{S1}U_{0}-1\right)\right]=\bar{U}$$

**Figure 1:**
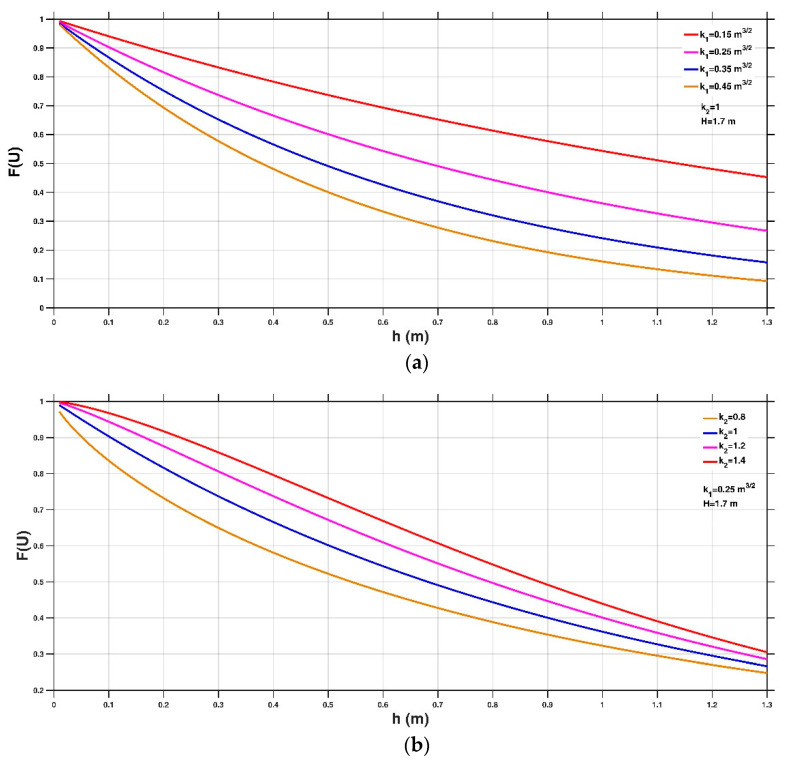
Distribution of cumulative distribution function (CDF) with respect to the water depth at different $k_{h1}$ (**a**) and $k_{h2}$ (**b**) values.

To formulate the incipient velocity of people during flood events U in the real (space) domain, a hypothesis on the CDF of U in the real (space) domain should be carried out to connect the probability domain to the real (space) domain. The hypothesized CDF should satisfy some characteristics of U: It is continuous and differentiable, it should vary from 0 to 1, and all of the values of U between $U_{0}$ and $U_{1}$ are equally likely. Some experiments have shown that the incipient velocity of people during flood events decreases rapidly with increasing water depth and is related to the friction force between people during flood events and the wet ground, the drag force exerted on the people by the flow, physical characteristics of people and physical properties of fluid (e.g., [6,8,9,10,11]). To this end, the following nonlinear CDF seems a good choice to satisfy the abovementioned characteristics:

(10) $$F\left(U\right)=\exp\left[-k_{h1}\sqrt{\frac{C_{d1}*\rho_{f}}{\mu_{1}*m_{H}}}{\left(\frac{h}{H}\right)}^{k_{h2}}\right]$$

where $k_{h1}$ and $k_{h2}$ are two empirical coefficients for the case of people instability ($k_{h1}$ has a unit of m^3/2^, whereas $k_{h2}$ is dimensionless), $\mu_{1}$ is the friction coefficient between the people during flood events and the wet ground, $C_{d1}$ is the drag coefficient exerted on the people by the flow, $\rho_{f}$ is the density of the surrounding fluid, $m_{H}$ and H are the mass and the height of a human body, respectively, and h is the water depth. Two terms, $\sqrt{\mu_{1}/C_{d1}}$ and $\sqrt{m_{H}/\rho_{f}}$, are introduced into Equation (10) based on the study of Xia et al. [6]. In their work, the incipient velocity formulae for the sliding instability and toppling instability of people in floodwaters were derived using a mechanics-based method. The hypothesized CDF also satisfies two main assumptions: (1) All values of between zero and infinity are equally likely and (2) the incipient velocity decreases from infinity at a very shallow water depth (limit case, h→0) to zero at a very deep water depth (another limit case, h→+∞). Taking constant values of $\mu_{1}$ = 0.65 and $C_{d1}$ = 1.55 from Keller and Mitsch [26] and Lind et al. [16] and $\rho_{f}$ = 1000 kg/m^3^ (clear floodwater), $m_{H}$ = 50 kg and H = 1.7 m as a typical example, Figure 1a,b shows the impacts of different $k_{h1}$ and $k_{h2}$ values on the hypothesized CDF. It can be seen that either an increasing $k_{h1}$ or a decreasing $k_{h2}$ leads to a smaller CDF value at a given water depth h.

Combining Equations (6) and (10) and using Equation (8) can yield an analytical expression for the incipient velocity U of people subject to flooding based on Shannon entropy theory:

(11) $$U=U_{0}+\frac{1}{\lambda_{S1}}\ln\left\{1+\left[\exp\left(\lambda_{S1}U_{1}-\lambda_{S1}U_{0}\right)-1\right]*\exp\left[-k_{h1}\sqrt{\frac{C_{d1}\rho_{f}}{\mu_{1}m_{H}}}{\left(\frac{h}{H}\right)}^{k_{h2}}\right]\right\}$$

## 3. Comparison with Experimental Data

To test the reliability of the proposed instability criterion (Equation (11)), seven experimental datasets available in the literature are collected and presented in this section, and a detailed summary regarding experimental setup, flow condition and tested people characteristics is provided in Table 1 based on Shand et al. [27], Russo et al. [28], and Gomariz et al. [29]. These experimental datasets contain different test subjects (children, adults, and human models) and various flow conditions and thus are representative of some human-flooding interaction systems.

Error estimation is carried out to quantitatively evaluate the accuracy of the derived entropy-based model against collected experimental datasets by calculating the correlation coefficient $R^{2}$ between the estimated and the observed datasets, the relative bias (RBIAS) between the estimated and the observed datasets, which is defined as *RBIAS* = $\frac{1}{N}\sum_{i=1}^{N}\left|\frac{m_{i}-o_{i}}{o_{i}}\right|$, and the root-mean-square error (RMSE), which is defined as *RMSE* = $\sqrt{\frac{1}{N}\sum_{i=1}^{N}{\left(m_{i}-o_{i}\right)}^{2}}$, where m and o are the estimated and observed data points, respectively, and N is the total number of observed data points, as also adopted by Zhu [30]. A larger $R^{2}$ value and smaller *RBIAS* and *RMSE* values indicate a model with a better goodness-of-fit.

**Figure 2:**
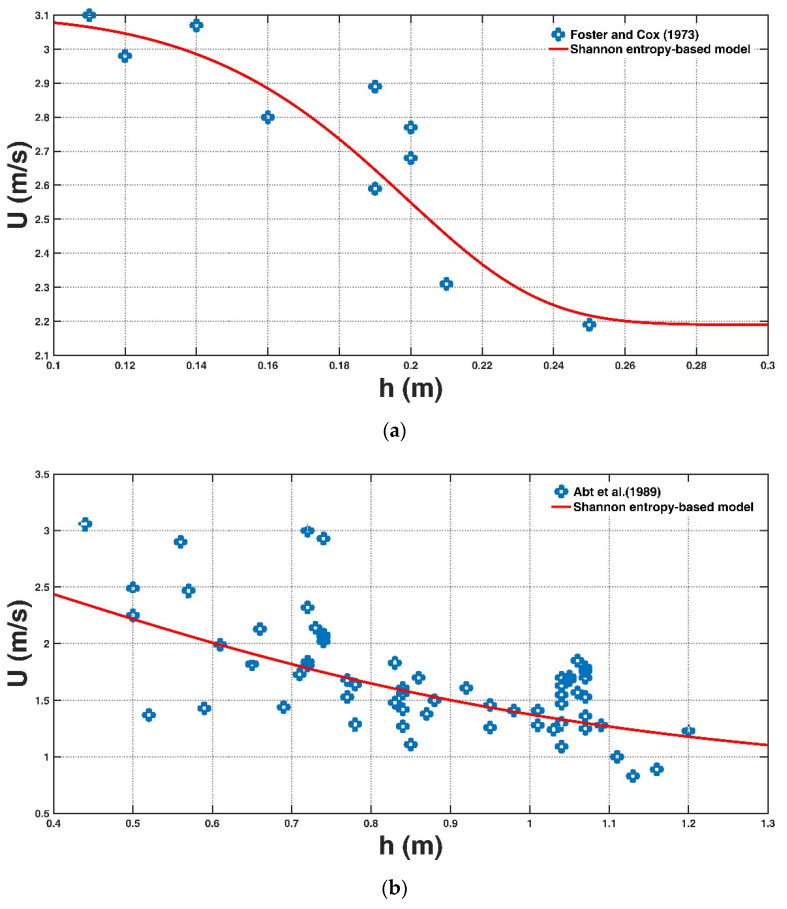

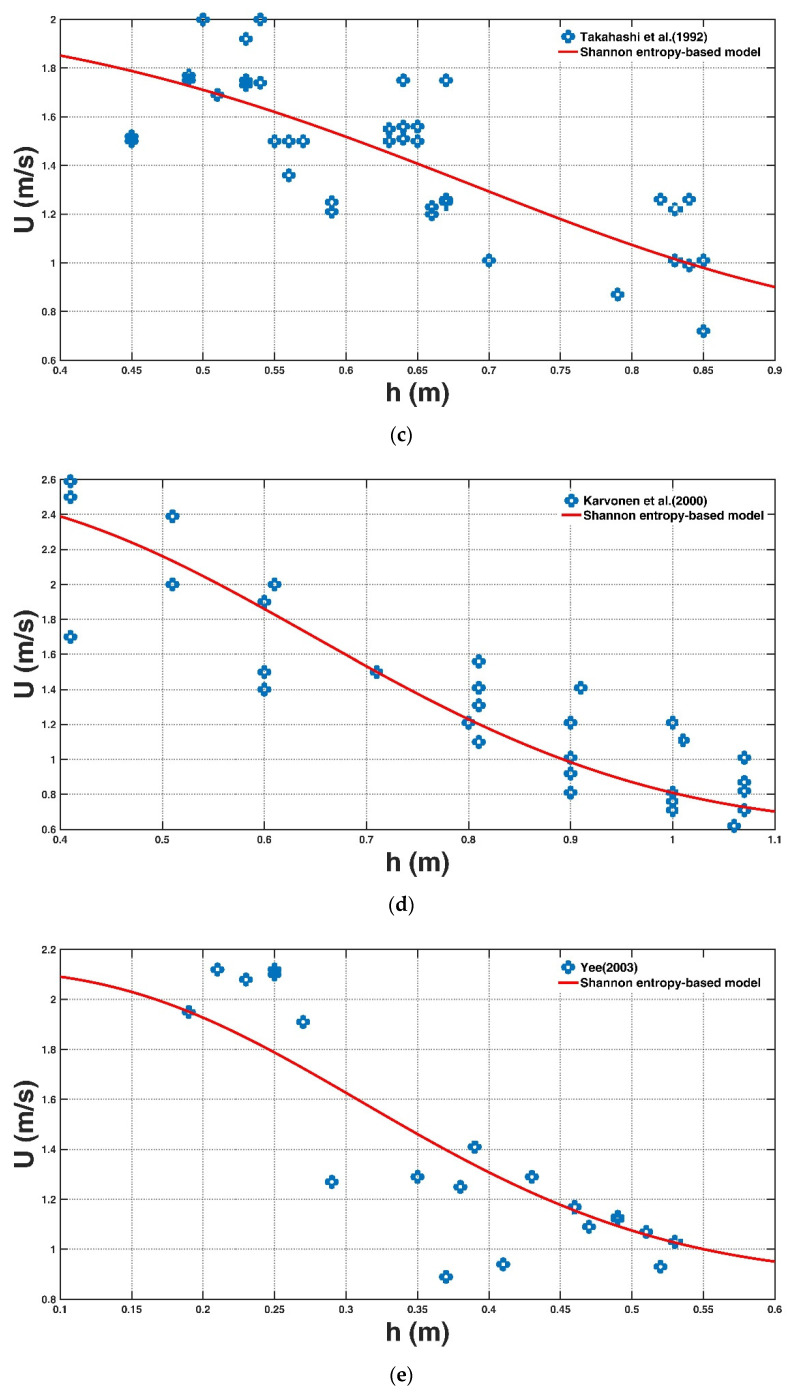

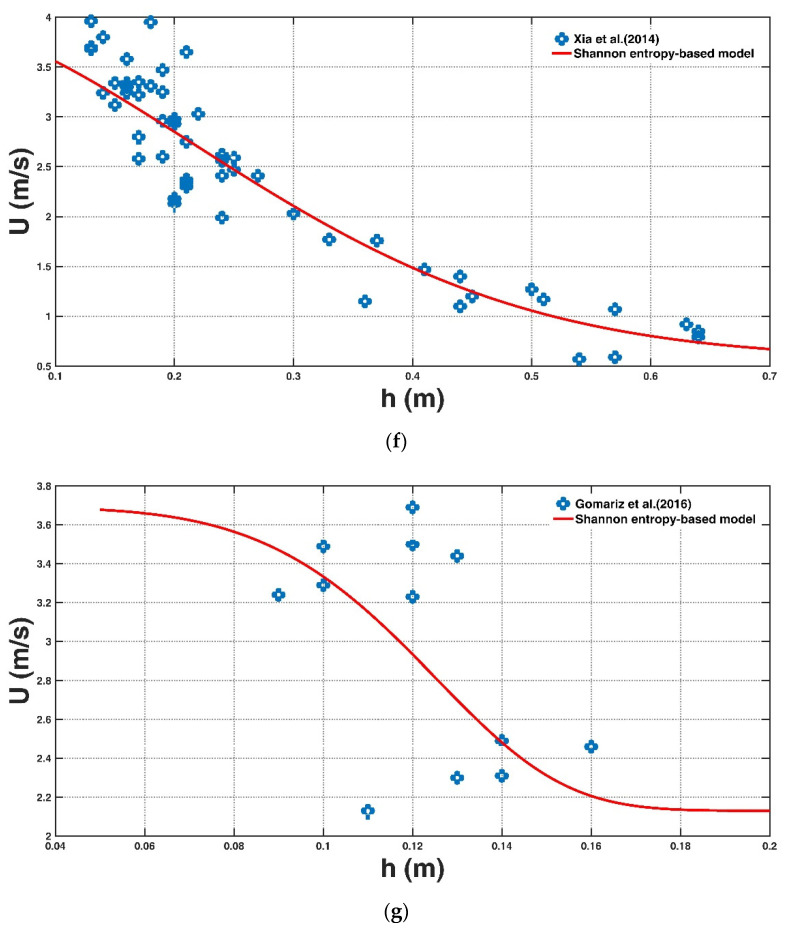
Comparison of the derived Shannon entropy-based model with experimental data points of Foster and Cox [14] (**a**), Abt et al. [11] (**b**), Takahshi et al. [15] (**c**), Karvonen et al. [12] (**d**), Yee [31] (**e**), Xia et al. [6] (**f**) and Gomariz et al. [29] (**g**).

For each experimental dataset, two Lagrange multipliers $\lambda_{S0}$ and $\lambda_{S1}$ are calculated by solving the nonlinear equation system in Equations (8) and (9). The parameters $U_{0}$, $U_{1}$ and $\bar{U}$ are provided from the experimental datasets, and the parameters $k_{h1}$ and $k_{h2}$ can be obtained by fitting Equation (10) to the experimental dataset. Figure 2a–g shows a comparison of the derived entropy-based model for each experimental dataset, and the calculated error parameters: $R^{2}$, *RBIAS,* and *RMSE*, are presented in Table 2. The proposed entropy-based model is consistent with the experimental results of Foster and Cox [14], Takahashi et al. [15], Karvonen et al. [12], Yee [30], and especially Xia et al. [6]. A deviation for some experimental datasets, including that of Abt et al. [11] (Figure 2b) and Gomariz et al. [29] (Figure 2g), is also noticeable, which is discussed as follows.

Abt et al. [11] examined the stability of 20 adults, including 18 males and two females, in a 61 m long, 2.44 m wide and 20 m deep laboratory flume. In their experiment, four kinds of bottom surfaces (steel, concrete, gravel, turf) and two groups of slopes (1:115, 1:38) were implemented. The measured water depth ranged from 0.43 m to 1.2 m, and the tested people experienced a flow velocity of 0.82–3.05 m/s. The underestimations of the estimated velocity with respect to the observed velocity might be associated with people adjusting their standing posture against the incoming floodwater in laboratory tests, whereas this effect is not taken into account in the proposed instability model, as pointed out by Xia et al. [6] and Milanesi et al. [17]. Chen et al. [9] proposed a new toppling instability criterion that considers the effect of leaning forward posture in floodwater, and the prediction accuracy for the experimental data of Abt et al. was improved [11]. Additionally, the measurement uncertainty of the instability test might also partly originate from different wearing styles of safety equipment and the subjective feelings of people towards instability.

In the study by Gomariz et al. [29], 16 women, five men and five children aged 6–55 years were tested for their instability in a physical flow model. Four kinds of shoes (heeled shoes, flat shoes, flip-flops, and waterproof boots) and three types of safety equipment (safety helmet, safety harness, and glasses to decrease visibility) were implemented. Compared with previous instability differentiations, these authors classified three degrees of flood hazards, including low hazard (small or inestimable instability), medium hazard (difficulty completing the test session), and high hazard (complete loss of stability). They also recorded the feelings of the tested people towards different flood hazard levels. The obvious deviation between the observed values and the measured values in Figure 2g might be associated with the variability in the psychological perception of the tested people when exposed to different degrees of flood hazard in the experiments, which is not taken into account in the proposed theoretical model. In addition, different wearing styles of shoes and safety equipment correspond to different friction coefficients and walking conditions in floodwaters, which is also not justified in the estimation based on Equation (11).

Thus, the proposed Shannon entropy-based model is feasible for the experimental datasets considering some scattering of experimental data points. This model could have a simple mathematical form and contain few calibrated coefficients. Furthermore, simple qualitative analysis results regarding some parameters that have been incorporated into the model can be in accordance with our understanding of the physical mechanism of the instability of people in floodwaters (as will be shown in Section 4). It is applicable to couple this entropic model with mature two-dimensional hydro-hydraulic models to mimic the flooding routing in terms of water velocity and water depth in an urban region, and its consequence to vulnerability of people in floodwaters (including adults and children, similar to the content in Section 5), provided that some empirical coefficients ($k_{h1}$,$k_{h2}$ and $\lambda_{S1}$) for real-life flooding events are calibrated from limited datasets. However, some limitations of the proposed data-driven entropic model also need to be pointed out in this study. For example, adjusting the posture for possible protection against the incoming floodwater is worthy of consideration in safety guideline formulation for people in floodwaters. However, the entropic model does not contain this effect.

## 4. Sensitivity Analysis

Taking some constant values of $\mu_{1}$ = 0.65, $C_{d1}$ = 1.55, $\rho_{f}$ = 1000 kg/m^3^, $m_{H}$ = 50 kg, H = 1.7 m, $k_{h1}$ = 0.25 m^3/2^ and $k_{h2}$ = 1 as examples [6,13,16,26], a qualitative analysis of the Shannon entropy-based model (Equation (11)) for the instability of people in floodwaters is performed in this section.

**Figure 3:**
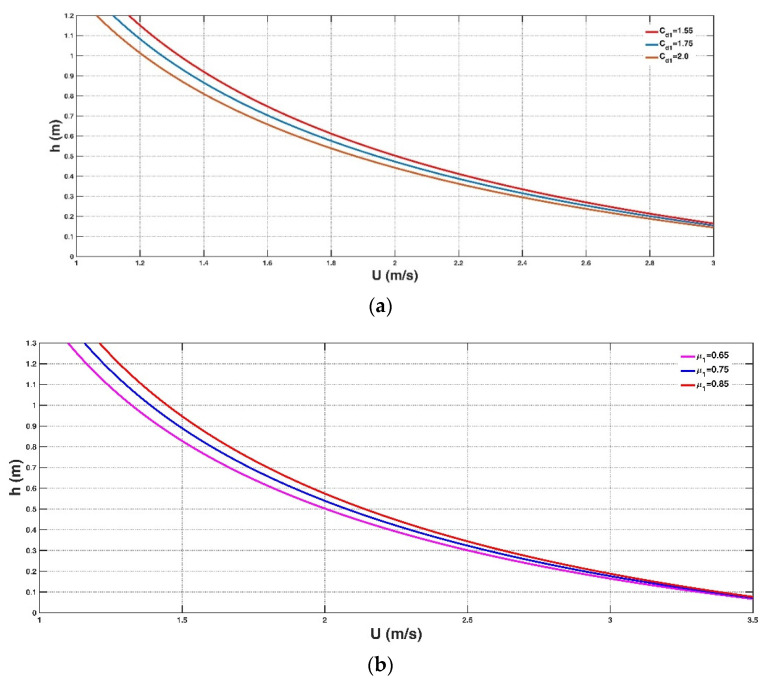

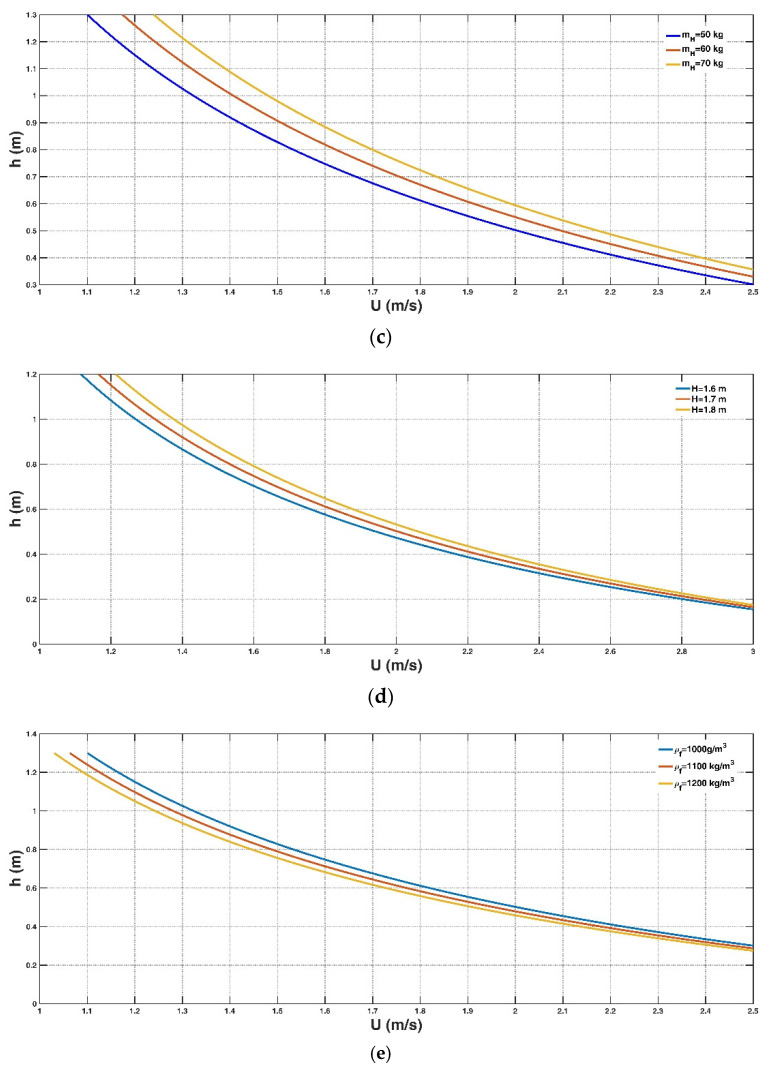
Qualitative analysis of some parameters of the Shannon entropy-based model for the instability of people in floodwaters (Equation (11)), including drag coefficient (**a**), friction coefficient (**b**), human body mass (**c**), and height (**d**), and surrounding fluid density (**e**).

Figure 3a–e shows the influence of some parameters on the Shannon entropy-based model for assessing instability (Equation (11)), including the drag coefficient $C_{d1}$, friction coefficient $\mu_{1}$, human body mass $m_{H}$ and height H and fluid density $\rho_{f}$. Figure 3a shows that strong floodwater weakens a person’s stability while increasing the friction force between the soles of shoes and wet ground during flood events can facilitate people’s safety as shown in Figure 3b. The value of the friction coefficient $\mu_{1}$ has a wide range depending on different circumstances in previous studies [17]. In the experiment of Takahashi et al. [15], friction coefficients were estimated corresponding to different combinations of soles of three persons and the ground surfaces: $\mu_{1}$ ranges from 0.38 to 1.49 in the case of wet ground, especially for the concrete surfaces filled with algae, $\mu_{1}$ is estimated to be 0.4, whereas for other surfaces, $\mu_{1}$ = 0.6 is recommended. In the experiment of Keller and Mitsch [26], the authors suggested a $\mu_{1}$ value of 0.3, whereas $\mu_{1}$ = 0.46 was estimated in the work of Milanesi et al. [17]. These experimental results have shown that the tested person could be more resistant to the incoming flow with a large friction effect between the sole and the ground surface. A heavy person is more likely to maintain stability than a light person due to the larger gravity (Figure 3c). Compared with a short person, a tall person could experience a lower buoyancy force, thereby improving their stability (Figure 3d). During flooding events, floodwaters always carry debris, trees, containers, etc. [13]. Consequently, the fluid density is improved, thus enlarging the buoyancy force of people during flood events and leading to an acceleration of instability, as shown in Figure 3e. These qualitative results are in accordance with our understanding of the physical mechanism of the instability of people in floodwaters.

## 5. Application of the Entropy-Based Model

**Figure 4:**
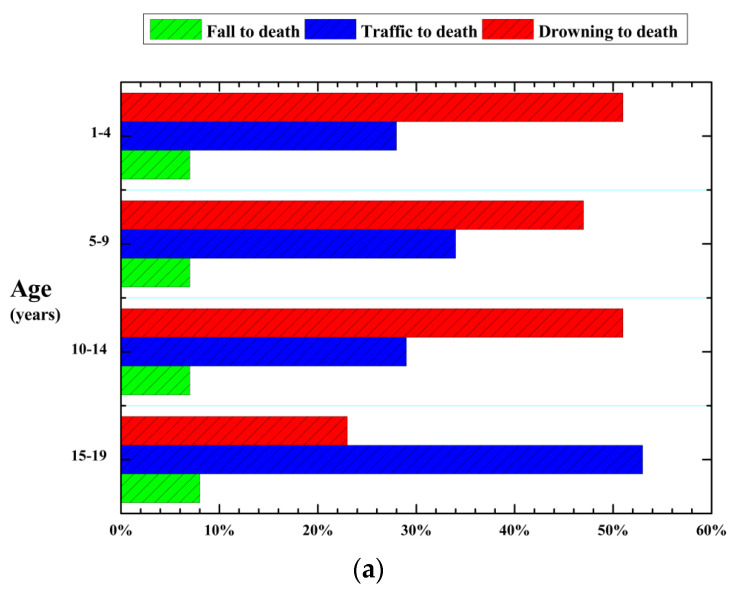

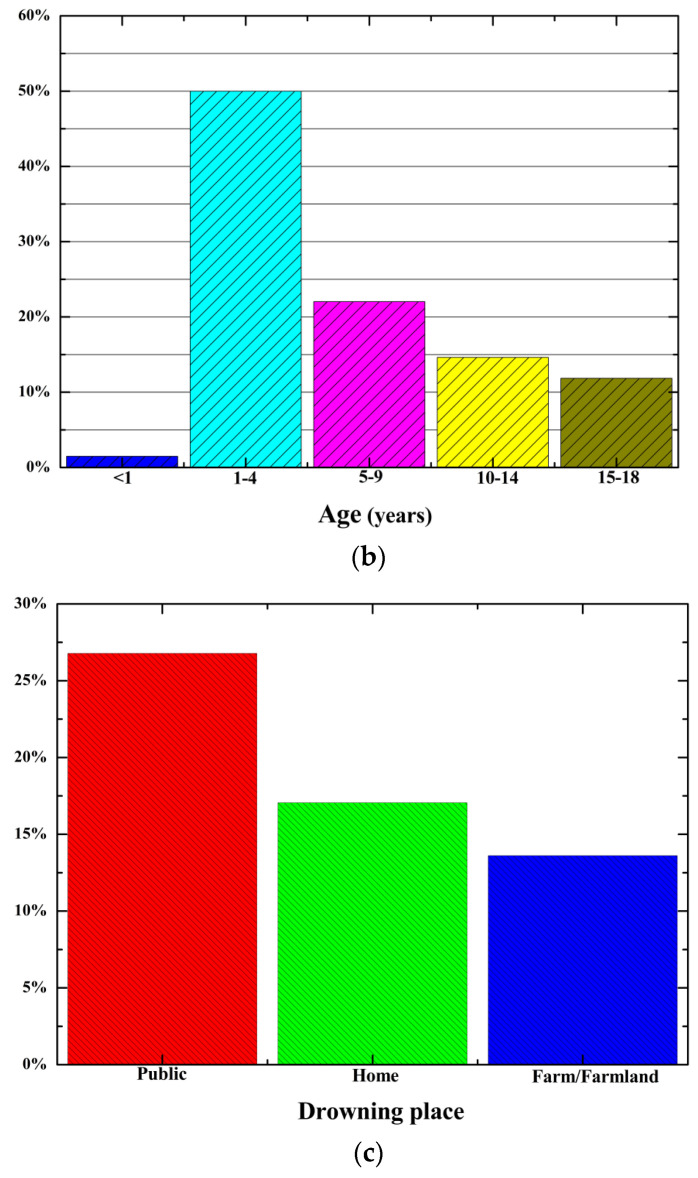
First three causes of death among Chinese adolescents and children aged 0–19 years from 2010 to 2015 (**a**) and age distribution (**b**) and location (**c**) of drowning cases in outpatient and emergency departments for Chinese adolescents and children aged 0–18 years. Data were obtained from the report “Injury Review of Chinese Adolescents and Children (2010–2015)”.

**Figure 5:**
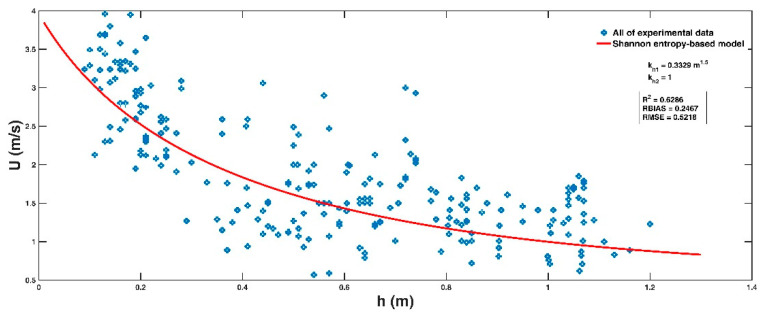
Comparison of the entropy-based model (Equation (11)) with all of the experimental data points in Section 3 for the estimation of $k_{h1}$ and $k_{h2}$.

Taking the physical parameters (height and mass) of Chinese adults and children as a representative example, this section endeavors to recommend safety guidelines for Chinese vulnerability to floodwaters. According to the report “Nutrition and Chronic Diseases in China (2015)” published by the National Health and Family Planning Commission of China in 2015, the average weight and height of men aged over 18 years old were 1.671 m and 66.2 kg, respectively, and those of women were 1.558 m and 57.3 kg, respectively. Regarding Chinese children, according to the report “Injury Review of Chinese Adolescents and Children (2010–2015)”, which was jointly published by the Chinese Centre for Disease Control and Prevention and Safe Kids Worldwide (China) in 2017, 54,194 adolescents and children aged 0 to 19 years died from injuries per year in China during 2010–2015, and drowning was the most important cause of death, especially for those aged 1–14 years old, as shown in Figure 4a. Among all drowning cases in the outpatient and emergency departments, children aged 1–4 years accounted for the highest proportion (49.99%) (Figure 4b) and 26.78% of drowning cases occurred in public areas (Figure 4c). Therefore, the following physical parameters of a 4-year-old boy and a 4-year-old girl were considered as representative examples in the flood hazard map: H = 1.041 m and $m_{H}$ = 16.64 kg for the boy and H = 1.031 m and $m_{H}$=16.17 kg for the girl based on statistics from the Chinese Children Physical Development. These data were included because of the psychological development of 4-year-old children, which may cause them to disregard parental advice and engage in risky behavior. Considering the serious situation of a child drowning in floodwaters, we set a drowning depth limit $H_{hm}$ in the safety guideline as implemented by Milanesi et al. [17]: The maximum water depth is assumed to be equal to the height of the head of a child from the ground, and Milanesi et al. [17] suggested that it was 13/16* H based on previous analyses [32]. Empirical values of $\mu_{1}$ = 0.65, $C_{d1}$ = 1.55, and $\rho_{f}$ = 1000 kg/m^3^ that have been adopted by some researchers [6,13,16,26] are also used here. Considering the heterogeneous nature of the collected experimental data, we calculated two values of empirical coefficients $k_{h1}$ and $k_{h2}$ by comparing the entropy-based model (Equation (11)) to all of the experimental data points in Section 3, as shown in Figure 5.

**Figure 6:**
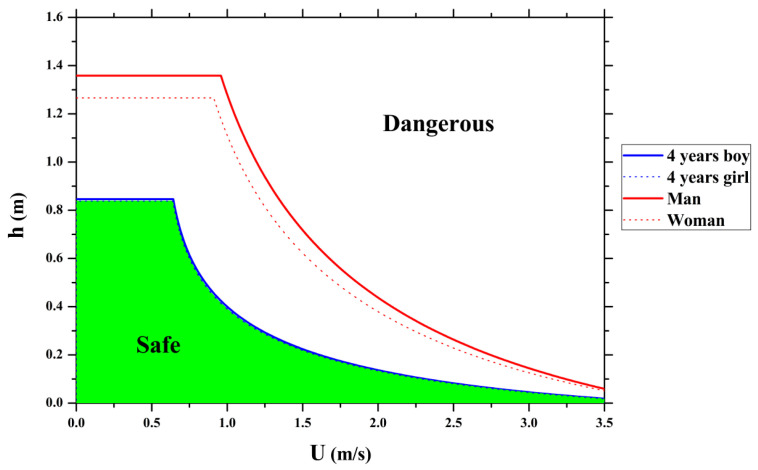
Vulnerability degree for Chinese adults and children exposed to flooding.

Figure 6 shows the vulnerability degree for Chinese adults and children exposed to urban flooding using Equation (11), which can be used as a reference by administrators and stakeholders for flood hazard mitigation and flood strategy management. The safety threshold for a 4-year-old child should be much lower than that for an adult. In this figure, the green and upper parts denote the safe region and the dangerous region for both Chinese adults and children, respectively, while the middle section between the above two shows the region where the child is in danger, but the adult is temporarily safe. The safety threshold for men is a little higher than that for women since a man is always heavier and taller than a woman, according to demographic statistics. With an increasing water level and an intensive flow velocity, the stability conditions of both adults and children are threatened, and more protective evacuation measures against floodwaters are needed. Additionally, it should be noted that the vulnerability degree for people in other regions could also be evaluated using Equation (11) as long as the demographic characteristics are provided.

## 6. Concluding Remarks

A number of cities worldwide have experienced serious consequences due to urban flooding in the context of global climate change and rapid urbanization. Investigations into the safety criteria of people during urban flooding events can assist in the implementation of disaster prevention and mitigation measures. In this study, the hydrodynamic instability criterion of people in floodwaters in terms of the incipient velocity and water depth were derived by the probability method based on the Shannon entropy theory. The derived entropy-based model was tested against seven experimental datasets available in the literature, and its validity was verified for some experimental data points considering a degree of data scattering. Furthermore, a sensitivity analysis of the derived model to some incorporated parameters was performed, and the qualitative results are in accordance with our understanding of the physical mechanism of the instability of people in floodwaters. Taking the physical parameters (height and mass) of Chinese adults and children as a representative example, this study showed the vulnerability degree for Chinese adults and children subject to floodwaters. These data could be used as a reference by administrators and stakeholders for flood hazard mitigation and flood strategy management.

## Figures and Tables

**Table 1:** Summary of collected experimental data regarding human instability in the literature based on Shand et al. [27], Russo et al. [28] and Gomariz et al. [29]. Caption: $k_{h1}^{’}=k_{h1}\sqrt{C_{d1}\rho_{f}/\mu_{1}m_{H}}$ is dimensionless.

Case		Foster and Cox [14]	Abt et al. [11]	Takahashi et al. [15]	Karvonen et al. [12]	Yee [31]	Xia et al. [6]	Gomariz et al. [29]
Experimental setup		Flume: 6 m long, 0.6 m wide, and 0.75 m deep	Flume: 61 m long, 2.44 m wide, and 1.22 m deep	Funneled basin: 50 m long and 20 m wide	Moving platform through basin: 130 m long, 11 m wide and 5.5 m deep	Flume	Flume: 60 m long, 1.2 m wide and 1 m deep	Same as in Russo (2009)
Flow condition	Slope	Horizontal	1:115; 1:38	Horizontal	Horizontal	Horizontal	Horizontal	0%, 2%, 4%, 6%, 8%, 10%
	Water depth (m)	0.09–0.41	0.43–1.2	0.44–0.93	0.4–1.1	0.18–0.53	0.02–0.13	0.1–0.18
	Flow velocity (m/s)	0.76–3.12	0.82–3.05	0.58–2	0.6–2.6	0.89–2.12	0.2–1.8	2.1–3.75
Tested people characteristics	Subject characteristics	Male children (9–13 years)	Adults with safety equipment (male and female)	Adult males	Adults: 5 males and 2 females	Children (6–8 years): 2 males and 2 female	Model human body	Adults (6–55 years): 5 males and 16 females; 5 children (<15 years)
	Number of tested subjects	6	20	3	7	4	8 (sliding), 46 (toppling)	26
	Height of tested people (m)	1.27–1.45	1.52–1.83	1.64–1.83	1.6–1.95	1.09–1.25	1.7 m refers to the prototype from the 0.3 m model	1.32–1.73
	Weight of tested people (kg)	25–37	41–91	63–73	48–100	19–25	60 kg refers to the prototype from the 0.334 kg model	37–71
	People action	Standing, walking, turning and sitting	Standing, turning and walking	Standing	Standing, turning and walking	Standing, walking	Standing	Standing, walking
	Failure mechanism	Subject feels unsafe or loses footing	Subject loses footing	Subject loses footing	Subject loses footing	Subject feels unsafe or loses footing	Sliding, toppling	Subject feels unsafe or loses footing

**Table 2:** Comparison result of Shannon entropy-based model for each experimental dataset.

Case		Foster and Cox [14]	Abt et al. [11]	Takahashi et al. [15]	Karvonen et al. [12]	Yee [31]	Xia et al. [6]	Gomariz et al. [29]
Comparison with the entropy-based model	$k_{h1}^{\prime }$	20,000	2.5	19	17	18	16	240,000
	$k_{h2}$	5	1.9	3.2	4	2.9	1.6	5
	*R* ^2^	0.8290	0.4291	0.5886	0.8134	0.7855	0.8716	0.2484
	*RBIAS*	0.0407	0.1606	0.1222	0.1451	0.1214	0.1292	0.1364
	*RMSE*	0.1258	0.3766	0.1991	0.2478	0.2166	0.3495	0.4921
